# Trimethylamine-N-oxide has prognostic value in coronary heart disease: a meta-analysis and dose-response analysis

**DOI:** 10.1186/s12872-019-01310-5

**Published:** 2020-01-09

**Authors:** Miao-En Yao, Peng-Da Liao, Xu-Jie Zhao, Lei Wang

**Affiliations:** 1 LongHua Hospital Shanghai University of Traditional Chinese Medicine, Shanghai, 200032 China; 2 Second Clinical College of Guangzhou University of Chinese Medicine, Guangzhou, 510405 China; 3 Department of Cardiovascular Medicine, 2nd Affiliated Hospital of Guangzhou University of Chinese Medicine, NO. 111 Dade Road, Yue-Xiu District, Guangzhou, 510120 China; 4 Department of Critical Care Medicine, Second Affiliated Hospital of Guangzhou University of Chinese Medicine, Guangzhou, 510120 China

**Keywords:** Trimethyloxamine, Coronary disease, Dose-response relationship, Meta-analysis as topic, Differential threshold

## Abstract

**Background:**

Previous clinical studies have suggested that trimethylamine-N-oxide (TMAO) could contribute to the development of atherosclerosis cardiovascular disease. However, the synthetic analysis in coronary heart disease (CHD) was not yet performed. We aimed to clarify the relationship between elevated plasma concentrations of TMAO and the incidence of major adverse cardiovascular events (MACE) in CHD patients.

**Methods:**

Meta-analysis and dose-response analysis of hazard ratio data from prospective observational studies reporting on the association between TMAO plasma concentrations and the incidence of MACE in patients with CHD were conducted.

**Results:**

Of the 2369 published articles identified in the search, seven papers, with data from nine cohort studies (10,301 patients), were included in the meta-analysis. Combined data showed that elevated plasma TMAO concentrations could increase 58% higher risk of MACE in patients with CHD (hazard ratios [HR]: 1.58; 95% confidence interval [CI] = 1.35–1.84, *P* = 0.000). For follow-up ≥ 1 year, it was associated with 62% higher risk of MACE in patients with longer-term than shorter-term (HR for follow-up ≥ 4 years: 1.96; 95% CI = 1.52–2.52 vs one to 3 years: 1.34; 95% CI = 1.26–1.43, *P* = 0.004). The dose-response analysis revealed a ‘J’ shaped association between TMAO concentration and the incidence of MACE (*P* = 0.033), with the concentration above 5.1 μmol/L being associated with HR of > 1.

**Conclusions:**

Elevated levels of TMAO are associated with an increased incidence of MACE in patients with CHD. TMAO concentration of 5.1 μmol/L may be a cut-off value for prognosis.

## Background

Coronary heart disease (CHD) has become the leading cause of death throughout the world in recent years [1, 2]. Hence, it is urgent to establish a precise system to track its development. In order to observe the progress of disease, it is significant to discover a biological marker used to predict the adverse events for CHD patients. As the accurate biomarkers of prognosis being of increasing clinical value, more studies [3, 4] evaluating their validity of prognostic value have been performed. Currently, a metabolite called trimethylamine-N-oxide (TMAO) has recently shown association with the incidence of major adverse cardiac events (MACE) in patients with CHD [5–11]. In food such as red meat and eggs, choline and carnitine are the most natural source of TMAO. Metabolized by bacteria in the intestine, choline and carnitine are formed into trimethylamine (TMA), which are then absorbed into the blood and transformed into TMAO by oxidizing flavin monooxygenase enzymes (FMOs) in liver [12, 13]. Moreover, fish is another source, which is naturally rich in the preformed state of TMAO [14–16].

Clinical epidemiology demonstrates a positive correlation between higher plasma TMAO concentrations and an increased incidence of MACE [5–10]. Recently, one study [11] shows no significant correlation between acute-phase TMAO level and the incidence of MACE. A previous meta-analysis reveals a positive dose-dependent association between TMAO concentration and higher risk of MACE [17]. However, for CHD patients, it has not yet an analysis of synthesis for the prognostic value of TMAO. Particularly, the dose-response relationship between TMAO and the hazard ratios (HR) of MACE in CHD patients is uncertainty. Furthermore, there was no study on the explicit concentration of TMAO above which will increase the risk of MACE. Therefore, we have combined the results from published clinical trials to evaluate the prognostic value of plasma TMAO concentrations for MACE in the setting of CHD. Moreover, we have elucidated detail of the dose-response relationship and identified a cut-off value.

## Methods

### Search strategy

We searched several electronic databases (PubMed, Embase, Web of Science, Cochrane Library, ClinicalTrials.gov, Chinese VIP Information [VIP], China National Knowledge Infrastructure [CNKI], SinoMed and Wanfang Databases) up to 7 November 2019 for prospective, observational clinical studies, reporting on the association between TMAO and MACE in patients with CHD. We used wide search terms (Additional file 1) concerning aspects of ‘TMAO’ and ‘CHD’. We followed standard criteria for conducting and reporting meta-analyses created by MOOSE (Meta analyses of Observational Studies in Epidemiology) [18] (Additional file 2).

### Study selection

Prospective, observational studies evaluating the association between TMAO and MACE were included if they were conducted in a population with CHD at baseline, including acute coronary syndrome (ACS) or chronic CHD (defined as a history of myocardial infarction, percutaneous coronary intervention [PCI], coronary artery bypass grafting [CABG], or confirmation through coronary angiography). Publications without detailed data were excluded. In each publication, relative risk estimates (HR, risk ratios, or odds ratio) for increments of one standard deviation, or at least two TMAO categories, must have been available.

Three or more TMAO categories were required (with either 95% confidence intervals [CIs] or information to calculate them) for the dose-response analysis. For this analysis, a quantitative measure of TMAO and the number of each group must also have been available.

When multiple publications were published from the same source study, we used the publication with larger sample size or more relative data.

### Data extraction and quality assessment

The following data from each study were extracted: the first author’s name, publication year, country of study conduct, sample size, patient characteristics (gender, age), follow-up period, TMAO plasma concentrations, collection times and quantification, TMAO exposure level, relative risk estimates and 95% CIs for at least two categories of TMAO.

In the dose-response analysis, the proportions of patients who experienced MACE, HRs, and 95% CIs for all categories of TMAO, were necessary. These data were not explicitly presented in every publication, so we sent emails to the authors to request this information and waited 2 months for the reply. After this time, we only included those publications where we could calculate HRs and 95% CIs using published methods [19].

Data were extracted by one author (M.E.Y), and were checked for accuracy by another author (P.D.L). Study quality was assessed using the Newcastle-Ottawa scale (NOS) [20], containing three items; selection, comparability, and outcome. This scale awards a maximum of nine stars, and in general, a score of more than seven stars is accepted to represent a high quality study, whereas studies scoring less than five stars cannot be taken into a meta-analysis.

### Data synthesis and analysis

Stata/SE 14.0 software was used to perform all statistical analyses. Summary HR and 95% CI were calculated for the risk of MACE associated with high concentrations of TMAO in comparison with low concentrations [6, 7, 9–11], or increments of one SD [5, 8], using a random-effects model. The high TMAO concentration group refers to the highest quartile [7, 9, 10], tertile [6] or the higher one [11], while the low concentration group refers to the lowest or lower one. We pooled the natural logarithm of HRs and weighted HRs according to the method of inverse variance, considering a *P* < 0.05 from a two tailed analysis to be statistically significant. Subgroup analyses for patients with ACS [6, 8–11] and chronic CHD [5, 7] were conducted. Taking follow-up time into account, subgroups analyses were also performed for a follow-up duration of in-hospital [10], one to 3 years [5, 8, 9] and ≥ 4 years [6, 7, 9, 11].

The non-linear dose-response analysis between TMAO and HR for MACE was evaluated using restricted cubic splines with three knots through the ‘mvmeta’ command of Stata [21]. A non-linear random-effects model was established by combining a generalized least-squares method with a multivariate maximum likelihood method [22]. If a two tailed *P* < 0.05 is calculated, a dose-response relationship is considered for curve nonlinearity. All categories of TMAO, HR level of TMAO, and number of patients per study and per group, were included in the model (Additional file 3). The relevant commands of Stata 14.0 were showed on Additional file 4.

Heterogeneity was assessed with Chi-squared, Q-tests, and I^2^ statistics [23]. In Q-tests, *P* > 0.1 was not considered indicative of heterogeneity. For I^2^ statistics, the degree of heterogeneity was measured per the conventional four levels: I^2^ = 0–25% (low), I^2^ = 25–50% (moderate), I^2^ = 50–75% (substantial), I^2^ = 75–100% (extreme). Because the heterogeneity was substantial (50% < I^2^ < 60%), a random-effects model was used for the meta-analysis. For sensitivity analysis, a fixed-effects model was conducted to test whether the heterogeneity or pooled estimates could represent a reverse association. Given that fewer than ten studies were included in our analysis, the potential publication bias could not be quantified by using the Begg’s and Egger’s test or the funnel plot.

## Results

### Study selection

**Fig. 1:**
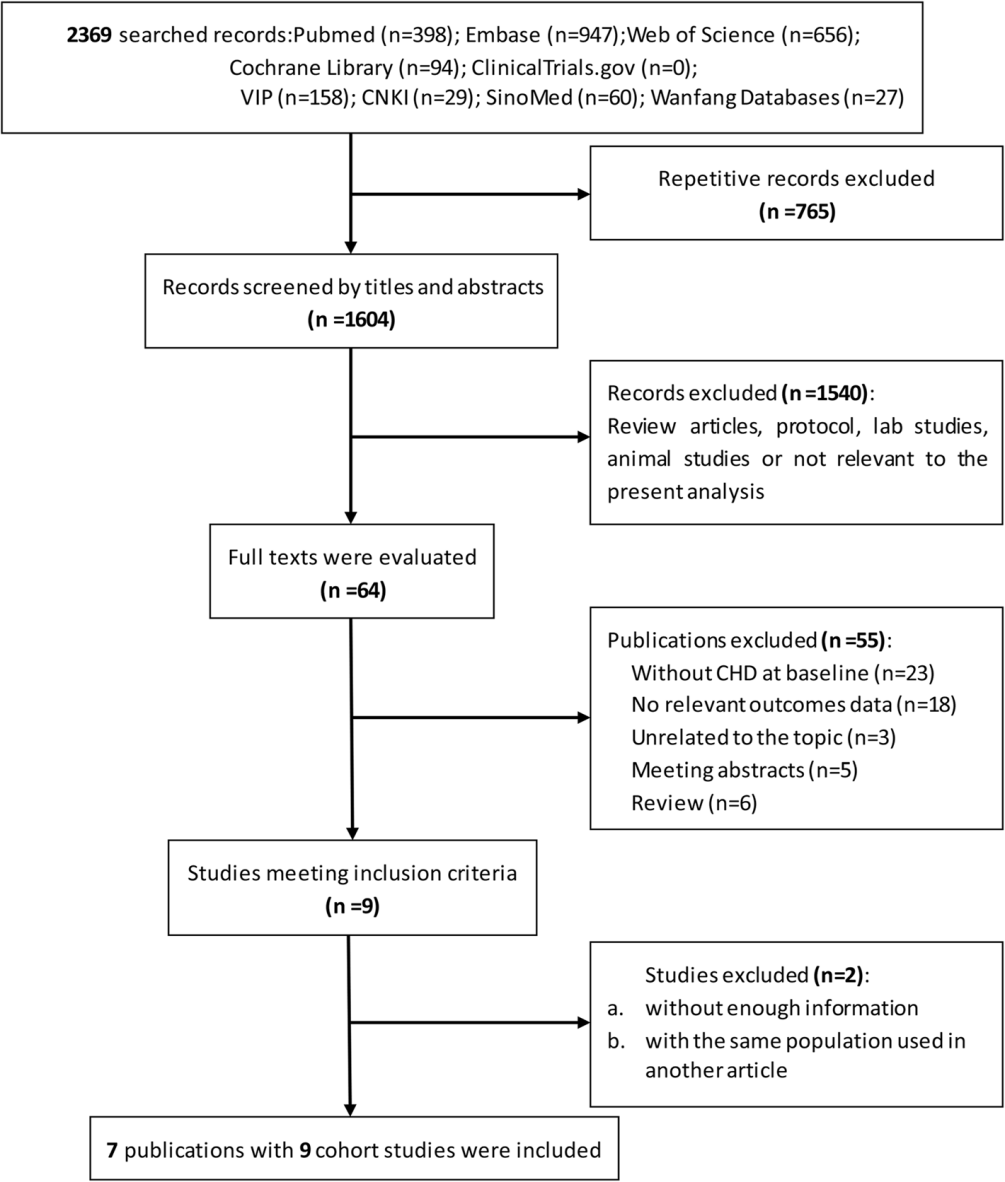
Flow diagram of study selection. CNKI, China National Knowledge Infrastructure; CHD, coronary heart disease; VIP, Chinese VIP Information

The search process and study selection (presented in Fig. 1) identified 2369 records of interest. Among these, 765 were repetitive and 1540 were excluded (confirmed via titles and abstracts) from the analysis because they were review articles, published protocol, lab studies, animal studies, or not of relevance. The full texts of 64 remaining articles were obtained. Several studies (Additional file 5) were subsequently excluded because they did not meet the predefined study inclusion criteria: studies of patients without CHD at baseline (23 papers), no relevant outcomes data (18 papers), unrelated topic (three papers), meeting abstracts (five papers), or reviews (six papers). A study by Wang Z. et al. [24] was excluded because of insufficient information on the inclusion criterion and follow-up period, and a second article by the same authors [25] was found to be a repetitive report from a partial dataset [5].

In total, seven publications, reporting on nine cohort studies, were selected for inclusion in the analysis [5–11]. A previous meta-analysis by Heianza Y. and colleagues was identified in the search results, but no additional and relevant publications were found in the reference list [26]. Among the included articles, one [6] reported on patients with and without diabetes separately, and one [9] reported on outcomes from two studies.

### Characteristics of included studies

Nine cohort studies, published from 2013 to 2019, reported on the relationship between the plasma concentration of TMAO and the incidence of MACE on patients with CHD. These studies are summarized in Table 1.

**Table 1:** Characteristics of included studies. (placed in the section of “result”, the first paragraph of “Characteristics of included studies”)

Study	Country	Age (mean ± SD)	Gender (% male)	Follow up (years)	Popula- tion	Samp- le size	TMAO^a^ (μmol/L)	TMAO type	Fast- ing	Quantification of TMAO	Collection time	HR (95% CI)	Outcomes	Model charac- ter	Adjust- ed	NOS			
																Selecti- on	Compar-ability	Outco-me	Total stars
Tang WH 2013 [5]	USA	63 ± 11	64	3	Chronic CHD	4007	3.7 (2.4–6.2)	plasma	+	LC/MS/MS	Time of cardiac catheterize- tion	1.30 (1.20–1.4, *P* < 0.001)	MACE	Per SD	+^b^	***	*	***	7
Lever M 2014 D [6]	New Zealand	74 ± 6.7	73	4.8	ACS	74	7.5 (4.4–12.1)	plasma	–	HPLC-MS/MS	Fourth-month post-discharge outpatient clinic visit	2.0 (1.1–3.6)	All cardiovascul- ar events	Tertile	–	***	*	**	6
Lever M 2014 UD [6]	New Zealand	68 ± 6.3	73	5.0	ACS	381	4.8 (3.0–9.1)	plasma	–			2.7 (1.6–4.8)	Death	Tertile	–	****	*	**	7
Senthong V 2016 [7]	USA	63 ± 11	71	5	Chronic CHD	2235	3.8 (2.5–6.5)	plasma	+	LC/MS/MS	Time of cardiac catheterize- tion, immediately prior to heparin injection and catheterizetion procedure	1.71 (1.11–2.61, *P* < 0.0138)	All-cause mortality	Quartile	+^c^	****	**	**	8
Suzuki T 2017 [8]	UK	67 ± 3.3	70	2	ACS	1079	3.7 (4.6–6.4)	plasma	–	LC-ToF-MRM	Days 1, 3 and 5 after admission	1.40 (1.26–1.55, *P* < 0.0005)	Death/MI	Per SD	–	****	**	***	9
CC Li XS 2017 [9]	USA	62.4± 13.9	57.5	7	ACS	530	4.28 (2.55–7.91)	plasma	–	LC/MS/MS	On presentation to the emergency room (baseline) and 4, 8 and 16 h later	1.81 (1.04–3.15, *P* < 0.05)	All-cause mortality	Quartile	+^d^	****	*	***	8
SC Li XS 2017 [9]	Swiss Confede-ration	63.9± 12.4	77.8	1	ACS	1683	2.87 (1.94–4.85)	plasma	–		Average time from the onset of chest pain to blood draw was 4.0 h	1.57 (1.03–2.41, *P* < 0.05)	MACE	Quartile	+^e^	***	*	***	7
Xu K-Z 2018 [10]	China	67.4± 12.8	44	in- hospital	ACS	200	5.9 ± 1.9	plasma	–	HPLC-MS/MS	At hospital admission or the next morning in fasting state	OR: 6.01 (2.03–17.73, *P* < 0.05)	MACE	Quartile	+^e^	***	**	***	8
Matsuzawa Y 2019 [11]	Japan	63.0± 2.5	88.0	5.4	ACS	112	5.63 (3.20–10.38)	plasma	–	LC-MS	At hospital admission before primary percutaneous coronary intervention	1.72 (0.63–4.92, *P* = 0.29)	cardiovascul- ar events	Above vs. below	+^f^	***	**	***	8

*ACS* acute coronary syndrome, *CHD* coronary heart diseases, *CC* Cleveland acute coronary syndrome cohort, *CI* confidence interval, *D* patients with diabetes, *F* female, *HR* hazard ratio, *HPLC-MS/MS* high performance liquid chromatography- tandem mass spectrometry, *LC-MS* liquid chromatography-mass spectrometry, *LC/MS/MS* stable isotope dilution high-performance liquid chromatography with on line electrospray ionization tandem mass spectrometry, *LC-ToF-MRM* stable-isotope dilution-hydrophilic interaction liquid chromatography-time of flight mass spectrometry with multiple reaction monitoring, *M* male, *MACE* major adverse cardiovascular event, *MI*myocardial infarction, *NOS* the Newcastle-Ottawa scale, *P P*-value, *SC* Swiss ACS cohort, *SD* standard deviation, *TMAO* trimethylamine-N-oxide, *UD* patients without diabetes, *UK* United Kingdom, *USA* United States of America

^a^Concentration of TMAO are presented as median (interquartile range) or mean ± standard deviation

^b^Adjusted for age, sex, smoking status, systolic blood pressure, low-density lipoprotein cholesterol level, high-density lipoprotein, cholesterol level, and status with respect to diabetes mellitus, and other baseline covariates

^c^Adjusted for age, sex, systolic blood pressure, diabetes mellitus, LDL, HDL, and smoking status, high sensitivity C reactive protein, estimated glomerular filtration rate, B-type natriuretic peptide, myeloperoxidase, number of diseased vessels, and medications

^d^Adjusted for age, gender, HDL, LDL, smoking, presence or absence of a history of diabetes mellitus, hypertension, hyperlipidemia, revascularization or coronary artery disease, C-reactive protein level, estimated glomerular filtration rate, initial troponin T level and diagnosis of either acute ST-segment elevation myocardial infarction (STEMI), non-STEMI or unstable angina

^e^Adjusted for age, left ventricular end-diastolic diameter, left ventricular ejection fraction, cardiac troponin I, high-sensitivity C-reactive protein, N-terminal pro-B-type natriuretic peptide and diabetes mellitus

^f^Adjusted for age, sex, hypertension, diabetes, dyslipidemia, smoking habits, systolic and diastolic blood pressure, triglycerides, high- and low-density lipoprotein cholesterol, glucose, hemoglobin A1c, estimated glomerular filtration rate, B-type natriuretic peptide, C-reactive protein, anterior myocardial infarction, atrial fibrillation, and medications on discharge

In total, the studies included 10,301 participants with an average age ranging from 62.4 to 74 years old. Three studies were conducted in the United States of America, two in New.

Zealand, one in Switzerland, one in The United Kingdom, one in China and one in Japan. Seven studies reported on patients with ACS while two included patients with chronic CHD. The TMAO concentration in plasma ranged from 2.87 to 7.5 μmol/L, with fasting samples being collected in two studies. The follow-up period used to calculate the HRs for MACE ranged from in-hospital to 7 years; three studies had a follow-up period of 1–3 years and five studies had a follow-up period of ≥4 years (Table 1). The defined outcomes were not the same in each study: some specifically looked at cardiovascular events or MACEs, whereas other looked at all-cause mortality. In several studies, the reported HR estimates were adjusted for variables such as age, gender, medication, disease, etc. [5, 7, 9–11]. The mean NOS score of all included studies was 7.56.

### The relationship between TMAO plasma concentration and the incidence of MACE in CHD patients

**Fig. 2:**
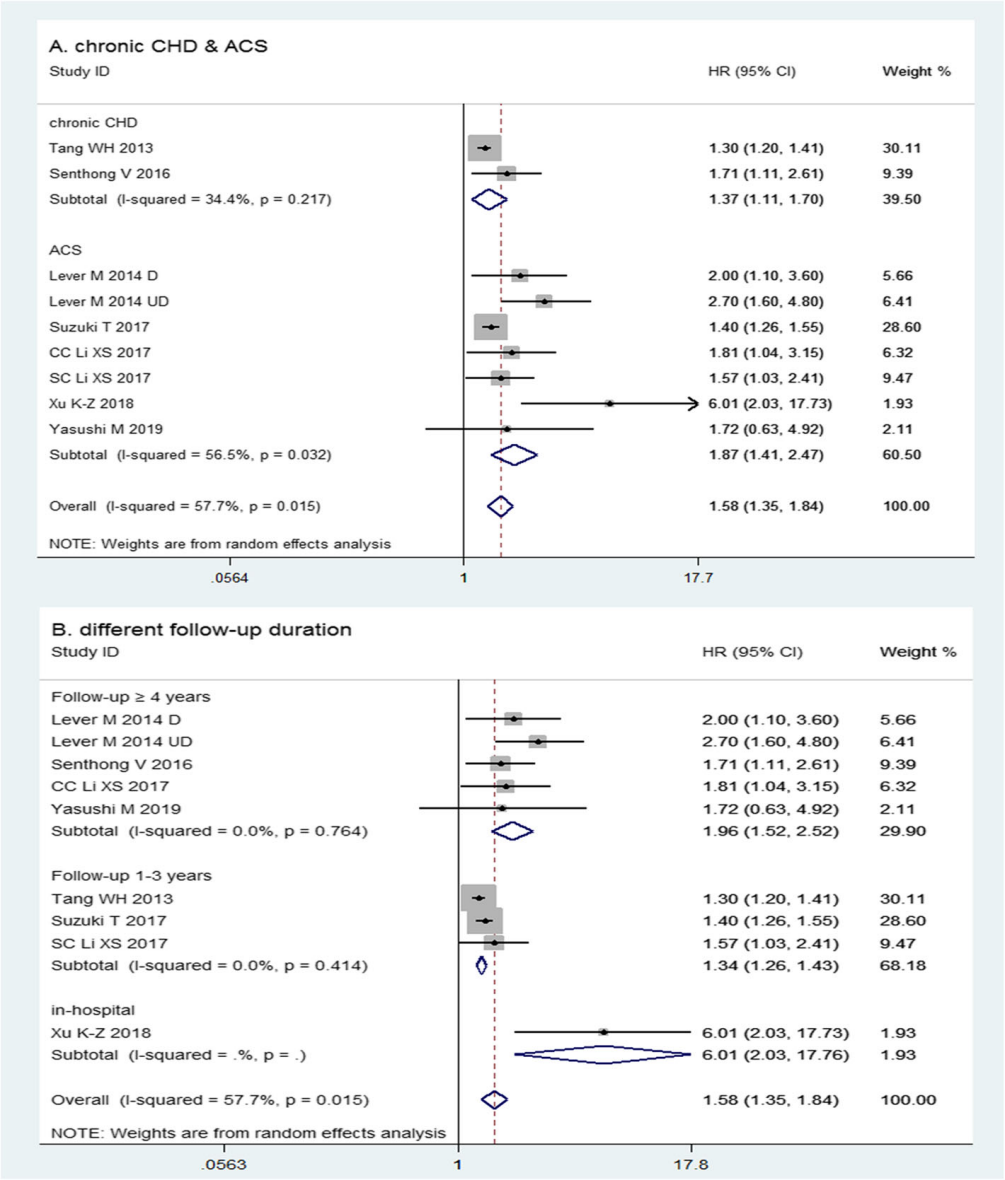
The relationship between TMAO plasma levels and incidence of MACE. In ACS and chronic CHD patients (**a**); in CHD patients with in-hospital observation and a follow-up of 1–3 years and ≥ 4 years (**b**). ACS, acute coronary syndrome; CC, Cleveland acute coronary syndrome cohort; CHD, coronary heart diseases; CI, confidence interval; HR, hazard ratio; MACE, major adverse cardiovascular event; SC, Swiss ACS cohort; TMAO, trimethylamine-N-oxide

The pooled HR estimates for MACE in patients with ACS [6, 8–11] and chronic CHD [5, 7] were 1.87 (95% CI = 1.41–2.47, *n* = 7, *P* < 0.001), and 1.37 (95% CI = 1.11–1.70, *n* = 2, *P* < 0.001; Fig. 2, Panel A; Additional file 6), respectively. The heterogeneity of the ACS (I^2^ = 56.5%) group was substantial and the chronic CHD group (I^2^ = 34.4%) moderate.

When stratified by the duration of the follow-up period, the pooled HR estimates for MACE were 1.34 (95% CI = 1.26–1.43, *n* = 3, *P* < 0.001) and 1.96 (95% CI = 1.52–2.52, *n* = 5, *P* < 0.001) in studies with 1–3 years [5, 8, 9] and ≥ 4 years follow-up [6, 7, 9, 11] (Fig. 2, Panel B; Additional file 6), respectively. Heterogeneity was similarly low in both groups (I^2^ = 0.0%). The in-hospital observation of one study [10] showed that HR estimate was 6.01 (95% CI = 2.03–17.76, *P* = 0.001) for MACE.

There was significant difference in pooled HR among groups with different durations of follow-up, where heterogeneity was also significantly different (*P* < 0.001). Heterogeneity and pooled HR were not significantly different between patients with ACS and chronic CHD (*P* = 0.058).

The pooled HR for MACE in all studies when comparing the highest plasma concentrations of TMAO with the lowest was 1.58 (95% CI = 1.35–1.84, *n* = 9, *P* < 0.001; Fig. 2; Additional file 6), with substantial heterogeneity (I^2^ = 57.7%) [5–11]. High TMAO plasma concentrations were associated with a significantly increased risk of MACE.

The results of sensitivity analysis were summarized in Additional file 6. No evidence of reverse HR estimates were found. Considering the substantial heterogeneity in total analysis and subgroup analysis of ACS, we left one study [10] by Xu K-Z et al. out. The pooled HR estimates were still significant and the heterogeneity decreased to moderation (Additional file 6).

### Dose-response analysis

The dose-response analysis used a total of twelve HR estimates, from three studies, with four concentrations of TMAO in each (Additional file 3).

**Fig. 3:**
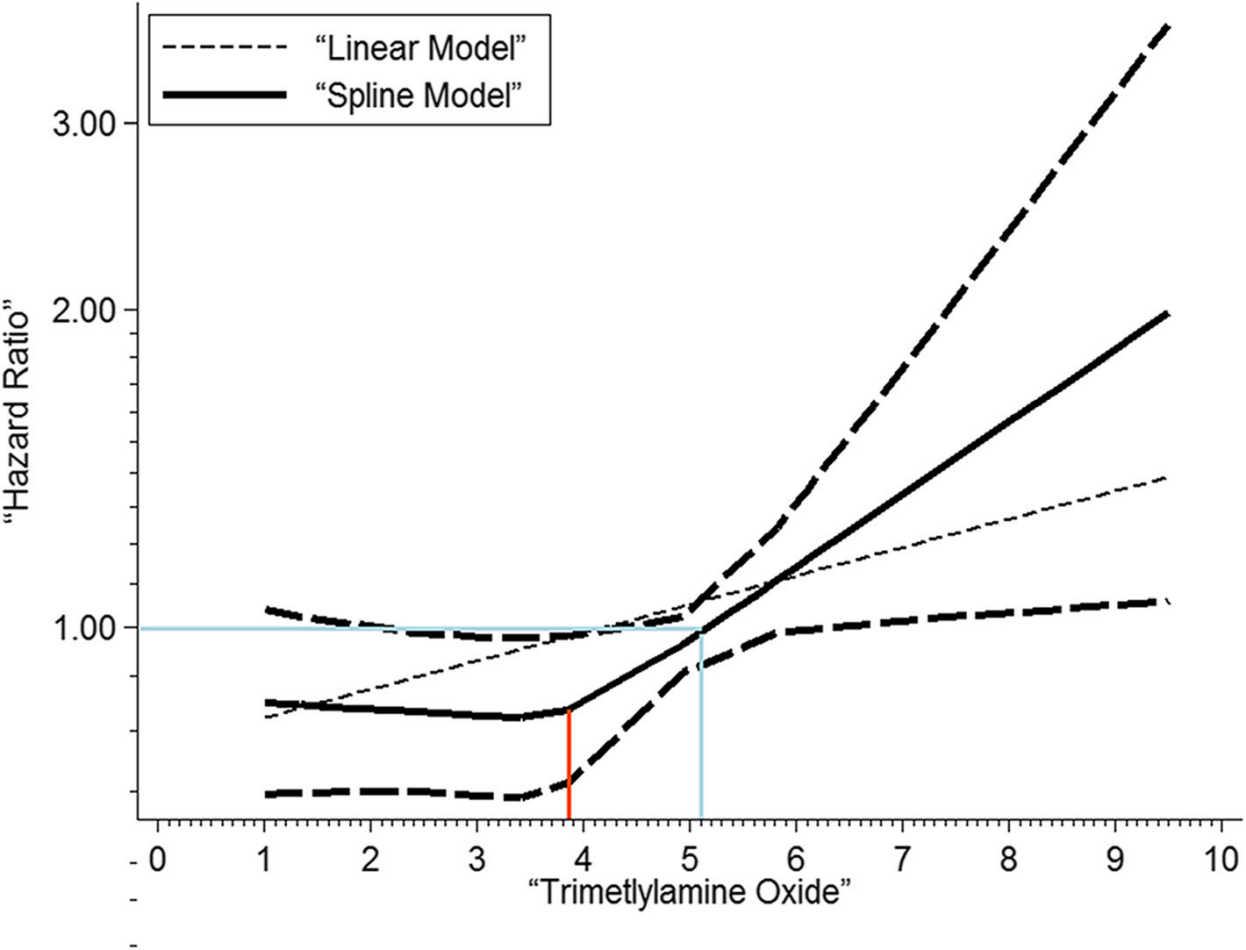
Concentration-risk analysis between plasma concentrations of TMAO and HR for MACE in patients with CHD. Used a restricted cubic splines in a non-linear random-effects model The solid line and the long dashed line represents the estimated HR and its 95% CI of the nonlinear relationship, while the short dashed line represents the linear relationship. Before the red line, the curve of HR tends to be flat, whereas after the blue line, the curve tends to be steep, and TMAO presents as a risk factor for MACE. CHD, coronary heart diseases; CI, confidence interval; HR, hazard ratio; MACE, major adverse cardiovascular event; TMAO, trimethylamine-N-oxide

This analysis revealed a ‘J’ shaped non-linear association between increasing TMAO concentration and increasing risk of MACE (*P* = 0.033; Fig. 3). As TMAO plasma concentrations rose above 3.9 μmol/L, the HR for MACE increased proportionally (red line). A HR > 1.0 was established above a concentration of 5.1 μmol/L (blue lines).

## Discussion

### Main findings

This meta-analysis evaluated the accumulating evidence for an association between higher TMAO plasma concentration and the incidence of MACE (defined as all cardiovascular events, death, all-cause mortality and myocardial infarction, et al.) in CHD patients. From the 2369 literature records identified, data from nine relevant cohort studies were included in the meta-analysis. We found that higher levels of plasma TMAO significantly increase the risk of MACE in patients with CHD, both in those with ACS and chronic CHD (HR 1.87 and 1.37, respectively), and over a short and long follow-up duration (HR 1.34 [1–3 years] and 1.96 [≥ 4 years], respectively). When the follow-up duration ≥ 1 year, we found significant heterogeneity of HR between groups with a longer and shorter term (*P* = 0.004), it may be that TMAO has a stronger prognostic value over the long term vs the short term. This may be partially caused by the accumulation effect of longer-term choline intake with more TMAO generating. The HR estimate (HR 6.01) was significantly highest for the in-hospital observation [10]; it may be caused by the acute stress damage, which may contribute to its heterogeneity. However, the identified studies only provided a ‘snapshot’ of TMAO, allowing little elucidation of the long term concentration profiles associated with the increased risk of MACE.

To date, no studies have identified the risk of MACE associated with specific plasma concentrations of TMAO. We conducted a dose-response analysis using data from three included studies. TMAO plasma concentrations less than 3.9 μmol/L were associated with low HRs for MACE (HR < 1), which increased almost linearly with increasing concentrations. This suggests that low TMAO concentrations (< 3.9 μmol/L) may even predict a lower incidence of MACE in CHD patients. TMAO was only associated with a HR > 1 when the concentration rose more than 5.1 μmol/L for populations with CHD. This suggests that 5.1 μmol/L may be a cut-off value. The result was in accordance with the median plasma TMAO concentrations of patients with MACE in two involved trials, the Cleveland Cohort study [9] (5.09 μmol/L) and the research performed by Tang WH et al. [5] (5.0 μmol/L). However, in the Swiss ACS Cohort study [9], the outcome was considered slightly higher, with lower median concentrations (3.75 μmol/L) in patients with MACE. More relevant prospective studies are needed to clarify the cut-off value of TMAO concentration for CHD patients.

### Potential mechanisms

Our dose-response analysis found that high plasma concentrations of TMAO (> 5.1 μmol/L) were associated with highly increased risk of MACE. A couple of experiments study showed that TMAO has the potential to accelerate the pathological progress by promoting atherogenesis, thrombosis, and vascular Inflammation [13, 27]. Cellular signaling studies also confirmed that TMAO could promote macrophage scavenger receptor expression, macrophage foam-cell formation [24, 28], platelet hyper-responsiveness to thrombosis [13, 29], and vascular inflammation [30–32]; all of which were relevant in the development of CHD and cardiovascular disease in general. Notably, in the animal experiments, the risk of atherosclerosis cardiovascular disease [24] and thrombosis [29] has increased as the plasma levels of TMAO raised. These results indicated that the dose-response relationship also existed in pathological changes, which could contribute to the incidence of MACE.

Furthermore, our analysis showed that TMAO may not be associated with a pathogenic process when the plasma concentration was under 3.9 μmol/L in patients with CHD. The nature of these processes is not yet clear. As a natural osmolyte, TMAO has a protein-stabilizing effect [33, 34] and has been shown to stabilize heavy meromyosin and the actomyosin complexes [35]. TMAO is also a chemical chaperone [36], and is capable of blunting the activation of the unfolded protein response, therefore limiting endoplasmic reticulum stress in injured cells, potentially during a cardiac injury. Interestingly, study in ApoE^−/−^ mice has also shown that TMAO can slow aortic lesion formation [37], suggesting that minor TMAO might have cardio protective effects in atherosclerosis.

Although the pathway for TMAO generation has been well established [12], the mechanism that control and drive increased concentrations in pathogenic scenarios is not well studied. Metabolism of a carbon (methyl) group in the amino acid betaine has been shown to indirectly increase TMAO generation [28], while increased activity of FMO3, a hepatic enzyme which transforms TMA into TMAO, could also increase TMAO concentrations [38]. So it is assumed that these associations could be causative, mechanisms to reduce these concentrations are of potential interest. According to a previous report [12], the concentration of TMAO can be reduced by limiting dietary choline intake, inhibiting or reducing the number of resident gut bacteria that promote TMAO generation, treating with probiotics, or restraining the activity of FMOs.

### Study strengths and limitations

Although meta-analysis of TMAO and the risk of MACE has been performed previously [17, 26], our study was the first to investigate the prognostic value of TMAO in the CHD population. Moreover, our dose-response analysis revealed key concentration thresholds that were associated with different levels of MACE risk in patients with CHD. This is a valuable knowledge in the future study of this association. Subgroup analyses added more detail to our findings, revealing that TMAO plasma concentrations had long term prognostic value.

The nine included studies were prospective and had an average NOS score of 7.56; failing to get stars mainly because they lacked demonstration of the outcome of interest, adequate control factors, or a statement of follow-up adequacy. There is potential for publication bias considering that no subgroups contain data from more than ten studies. Although the HRs identified from two studies [6, 8] were unadjusted by risk factors, there was no obvious heterogeneity in either the total, subgroup, or dose-response analyses. We found no evidence of reverse causation, and overall, we considered the analyses to be robust.

A limitation of the meta-analysis was that we found nine relevant studies to include, with only three contributing to the dose-response analysis. This limits our ability to summarize ‘real-world’ relative risk with precision. Furthermore, there were several differences in the methodology of these studies. TMAO concentrations can be influenced by several patient characteristics, such as diet (including regional differences), renal function, liver function, excretory function of gut [39, 40], or general condition of the patient, and also procedural factors, such as the plasma collection time. Differences in study methodology, such as follow-up duration, definition of MACE, and HR calculation were also significant sources of potential inaccuracy. The addition of new evidence to the field will significantly reduce the effect of these limitations in any potential future analyses.

Further studies should investigate the method to regulate the generation of TMAO, such as modulating intestinal flora, inhibiting the activity of FMOs [12]. In addition, more is required on the physiological function of TMAO in CHD patients and healthy individuals.

## Conclusion

This meta-analysis revealed a significant association between higher plasma TMAO concentrations and the long term risk of MACE in CHD patients, including those with ACS and chronic CHD. Our dose-response analysis found a non-linear relationship between TMAO concentration and the HR for MACE, with a concentration above 5.1 μmol/L being associated with a HR of > 1. Our findings are based on limited study data but clearly suggest that TMAO concentration has prognostic value for patients with CHD. More prospective researches are needed to evaluate this relationship and the mechanisms that drive it.

## Supplementary information

**Additional file 1.** Literature searches.

**Additional file 2.** MOOSE Checklist.

**Additional file 3.** Data used in the ‘TMAO concentration-risk of MACE’ analysis.

**Additional file 4.** Dose-response analysis by Stata/SE 14.0 (commands).

**Additional file 5.** Summary of 57 disqualified papers by evaluating full texts.

**Additional file 6.** Summarized results on the relative risk of MACE of elevated TMAO in patients with CHD.

## Data Availability

The datasets used and analyzed during the current study are available from the corresponding author on reasonable request.

## Abbreviations

ACS: Acute coronary syndrome
CHD: Coronary heart disease
CI: Confidence interval
CNKI: China National Knowledge Infrastructure
FMOs: Flavin monooxygenase enzymes
HR: Hazard ratios
MACE: Major adverse cardiovascular events
MOOSE: Meta analyses of observational studies in epidemiology
NOS: Newcastle-Ottawa scale
TMA: Trimethylamine
TMAO: Trimethylamine-N-oxide
VIP: Chinese VIP information
