# Supplementary material for: Trimethylamine-N-oxide has prognostic value in coronary heart disease: a meta-analysis and dose-response analysis
Source: BMC Cardiovasc Disord. 2020 Jan 9;20:7. doi: 10.1186/s12872-019-01310-5 (PMC6953212; doi:10.1186/s12872-019-01310-5)
Supplement: Supplementary file 1 — Additional file 1. Literature searches. [file 12872_2019_1310_MOESM1_ESM.docx]

**Additional file 1. Literature searches.**

1. **PubMed search**

((((((((((((((((((((((((((((((((((((((((((((((((((atherosis) OR Atherosclerosis) OR Coronary Artery Disease) OR Atheroscleroses) OR Atherogenesis) OR Artery Disease, Coronary) OR Artery Diseases, Coronary) OR Coronary Artery Diseases) OR Disease, Coronary Artery) OR Diseases, Coronary Artery) OR Coronary Arteriosclerosis) OR Arterioscleroses, Coronary) OR Coronary Arterioscleroses) OR Atherosclerosis, Coronary) OR Atheroscleroses, Coronary) OR Coronary Atheroscleroses) OR Coronary Atherosclerosis) OR Arteriosclerosis, Coronary) OR Artery Disease, Carotid) OR Artery Diseases, Carotid) OR Carotid Artery Disease) OR Carotid Artery Disorders) OR Artery Disorder, Carotid) OR Artery Disorders, Carotid) OR Carotid Artery Disorder) OR Disorders, Carotid Artery) OR Arterial Diseases, Carotid) OR Arterial Disease, Carotid) OR Carotid Arterial Disease) OR Carotid Arterial Diseases) OR Carotid Atherosclerosis) OR Carotid Atheroscleroses) OR Carotid Atherosclerotic Disease) OR Atherosclerotic Diseases, Carotid) OR Carotid Atherosclerotic Diseases) OR Atherosclerotic Disease, Carotid) OR Internal Carotid Artery Diseases) OR Arterial Diseases, Internal Carotid) OR Arterial Diseases, Common Carotid) OR Common Carotid Artery Diseases) OR External Carotid Artery Diseases) OR Arterial Diseases, External Carotid)) OR (((((((((((Cholesterol, VLDL) OR VLDL Cholesterol) OR Pre-beta-Lipoprotein Cholesterol) OR Cholesterol, Pre-beta-Lipoprotein) OR Pre beta Lipoprotein Cholesterol) OR Very Low Density Lipoprotein Cholesterol) OR Prebetalipoprotein Cholesterol) OR Cholesterol, Prebetalipoprotein) OR Cholesterol) OR Epicholesterol) OR cholesterin)) OR ((((Triglycerides) OR Triacylglycerol) OR Triacylglycerols) OR triglyceride)) OR (((((((((((((((((((oxidized low density lipoprotein) OR oxidized LDL) OR OxLDL) OR ox-LDL) OR electronegative low-density lipoprotein) OR LDL) OR minimally modified oxidized-LDL) OR MM-LDL) OR low density lipoprotein triglyceride) OR triacylglycerol LDL) OR LDL triacylglycerol) OR lipoprotein, LDL triglyceride) OR LDL triglyceride) OR very low density lipoprotein triglyceride) OR VLDL triglyceride) OR triglyceride VLDL) OR VLDL triacylglycerol) OR lipoprotein, VLDL triglyceride) OR triacylglycerol VLD)) OR ((((((((Hyperlipidemias) OR Hyperlipemia) OR Hyperlipemias) OR Hyperlipidemia) OR Lipidemia) OR Lipidemias) OR Lipemia) OR Lipemias)) OR ((((((((((((((((((((((((((((((((((((((((((((((((((((((((((Inferior Wall Myocardial Infarction) OR Anterior Wall Myocardial Infarction) OR Non-ST Elev ated Myocardial Infarction) OR ST Elevation Myocardial Infarction) OR Myocardial Infarction) OR Infarction, Myocardial) OR Infarctions, Myocardial) OR Myocardial Infarctions) OR Cardiovascular Stroke) OR Cardiovascular Strokes) OR Stroke, Cardiovascular) OR Strokes, Cardiovascular) OR Heart Attack) OR Heart Attacks) OR Myocardial Infarct) OR Infarct, Myocardial) OR Infarcts, Myocardial) OR Myocardial Infarcts) OR Myocardial Infarction, Inferior Wall) OR Diaphragmatic Myocardial Infarction) OR Diaphragmatic Myocardial Infarctions) OR Infarction, Diaphragmatic Myocardial) OR Infarctions, Diaphragmatic Myocardial) OR Myocardial Infarction, Diaphragmatic) OR Myocardial Infarctions, Diaphragmatic) OR Inferior Myocardial Infarction) OR Infarction, Inferior Myocardial) OR Infarctions, Inferior Myocardial) OR Inferior Myocardial Infarctions) OR Myocardial Infarction, Inferior) OR Myocardial Infarctions, Inferior) OR Acute Inferior Myocardial Infarction) OR Myocardial Infarction, Anterior Wall) OR Anterolateral Myocardial Infarction) OR Anterolateral Myocardial Infarctions) OR Infarction, Anterolateral Myocardial) OR Infarctions, Anterolateral Myocardial) OR Myocardial Infarction, Anterolateral) OR Myocardial Infarctions, Anterolateral) OR Anteroseptal Myocardial Infarction) OR Anteroseptal Myocardial Infarctions) OR Infarction, Anteroseptal Myocardial) OR Infarctions, Anteroseptal Myocardial) OR Myocardial Infarction, Anteroseptal) OR Myocardial Infarctions, Anteroseptal) OR Acute Anterior Wall Myocardial Infarction) OR Non ST Elevated Myocardial Infarction) OR NSTEMI) OR Non-ST-Elevation Myocardial Infarction) OR Infarction, Non-ST-Elevation Myocardial) OR Infarctions, Non-ST-Elevation Myocardial) OR Myocardial Infarction, Non-ST-Elevation) OR Myocardial Infarctions, Non-ST-Elevation) OR Non ST Elevation Myocardial Infarction) OR Non-ST-Elevation Myocardial Infarctions) OR ST Segment Elevation Myocardial Infarction) OR ST Elevated Myocardial Infarction) OR STEMI)) OR (((((((ACS) OR Acute Coronary Syndrome) OR Acute Coronary Syndromes) OR Coronary Syndrome, Acute) OR Coronary Syndromes, Acute) OR Syndrome, Acute Coronary) OR Syndromes, Acute Coronary)) OR (((((((((((((((((Heart) OR Hearts) OR Cardiovascular Disease) OR Disease, Cardiovascular) OR Diseases, Cardiovascular) OR cardiovascular disease) OR cardiovascular diseases) OR cardio disease) OR cardio) OR Heart Diseases) OR Disease, Heart) OR Diseases, Heart) OR Heart Disease) OR Cardiac Diseases) OR Cardiac Disease) OR Disease, Cardiac) OR Diseases, Cardiac)) OR (((((((((((CHD) OR Coronary Disease) OR Coronary Diseases) OR Disease, Coronary) OR Diseases, Coronary) OR Coronary Heart Disease) OR Coronary Heart Diseases) OR Disease, Coronary Heart) OR Diseases, Coronary Heart) OR Heart Disease, Coronary) OR Heart Diseases, Coronary)

**AND**

((((trimethyloxamine) OR trimethylammonium oxide) OR trimethylamine N-oxide) OR TMAO) OR trimethylamine oxide

1. **Embase search**

'coronary artery disease'/exp OR 'ischemic heart disease'/exp OR 'acute heart infarction'/exp OR 'chd' OR 'coronary diseases' OR 'disease, coronary' OR 'diseases, coronary' OR 'coronary heart diseases' OR 'disease, coronary heart' OR 'diseases, coronary heart' OR 'heart disease, coronary' OR 'heart diseases, coronary' OR 'ischemic heart disease' OR 'coronary disease' OR 'multivessel coronary artery disease' OR 'coronary artery insufficiency' OR 'coronary heart disease' OR 'coronary insufficiency' OR 'coronary occlusive disease' OR 'heart disease, ischaemic' OR 'heart disease, ischemic' OR 'ischaemia heart disease' OR 'ischaemic cardiac disease' OR 'ischaemic cardial disease' OR 'ischaemic cardiopathy' OR 'ischaemic heart disease' OR 'ischemia heart disease' OR 'ischemic cardiac disease' OR 'ischemic cardial disease' OR 'ischemic cardiopath' OR 'acute heart infarction' OR 'acute cardiac infarction' OR 'acute myocardial infarction' OR 'heart infarction, acute' OR 'cardiovascular tissue'/exp OR 'heart'/exp OR 'heart' OR 'cardiac effect'/exp OR 'cardiac effect' OR 'hearts' OR 'diseases, cardiovascular' OR 'cardiovascular disease'/exp OR 'cardiovascular disease' OR 'cardio disease' OR 'cardio' OR 'disease, heart' OR 'cardiac tissue'/exp OR 'cardiac tissue' OR 'cor'/exp OR 'cor' OR 'heart tissue'/exp OR 'heart tissue' OR 'myocardial tissue'/exp OR 'myocardial tissue' OR 'angiocardiopathy'/exp OR 'angiocardiopathy' OR 'angiocardiovascular disease'/exp OR 'angiocardiovascular disease' OR 'cardiovascular complication'/exp OR 'cardiovascular complication' OR 'cardiovascular diseases'/exp OR 'cardiovascular diseases' OR 'cardiovascular disorder'/exp OR 'cardiovascular disorder' OR 'cardiovascular disturbance'/exp OR 'cardiovascular disturbance' OR 'cardiovascular lesion'/exp OR 'cardiovascular lesion' OR 'cardiovascular syndrome'/exp OR 'cardiovascular syndrome' OR 'cardiovascular vegetative disorder'/exp OR 'cardiovascular vegetative disorder' OR 'complication, cardiovascular'/exp OR 'complication, cardiovascular' OR 'disease, cardiovascular'/exp OR 'disease, cardiovascular' OR 'major adverse cardiovascular event'/exp OR 'major adverse cardiovascular event' OR 'diseases, heart' OR 'heart disease'/exp OR 'heart disease' OR 'cardiac diseases' OR 'disease, cardiac' OR 'diseases, cardiac' OR 'cardio-vascular tissue'/exp OR 'cardio-vascular tissue' OR 'cardiac anomaly'/exp OR 'cardiac anomaly' OR 'cardiac disease'/exp OR 'cardiac disease' OR 'cardiac disturbance'/exp OR 'cardiac disturbance' OR 'cardiopathy'/exp OR 'cardiopathy' OR 'heart deficiency'/exp OR 'heart deficiency' OR 'heart deformity'/exp OR 'heart deformity' OR 'heart diseases'/exp OR 'heart diseases' OR 'heart disorder'/exp OR 'heart disorder' OR 'heart dysfunction'/exp OR 'heart dysfunction' OR 'major adverse cardiac event'/exp OR 'major adverse cardiac event' OR 'non st segment elevation acute coronary syndrome'/exp OR 'acute coronary syndrome'/exp OR 'acute coronary syndromes' OR 'acute coronary syndrome' OR 'non st segment elevation acute coronary syndrome' OR 'acs' OR 'coronary syndrome, acute' OR 'coronary syndromes, acute' OR 'syndrome, acute coronary' OR 'syndromes, acute coronary' OR ('inferior myocardial infarction'/exp OR 'anterior myocardial infarction'/exp OR 'acute heart infarction'/exp OR 'heart infarction'/exp OR 'non st segment elevation myocardial infarction'/exp OR 'posterior myocardial infarction'/exp OR 'silent myocardial infarction'/exp OR 'st segment elevation myocardial infarction'/exp OR 'heart rehabilitation' OR 'anterior myocardial infarction' OR 'inferior wall myocardial infarction' OR 'anterior wall myocardial infarction' OR 'non-st elev ated myocardial infarction' OR 'st elevation myocardial infarction'/exp OR 'st elevation myocardial infarction' OR 'myocardial infarction'/exp OR 'myocardial infarction' OR 'infarction, myocardial' OR 'infarctions, myocardial' OR 'myocardial infarctions' OR 'cardiovascular stroke' OR 'cardiovascular strokes' OR 'stroke, cardiovascular' OR 'strokes, cardiovascular' OR 'heart attack: heart infarction' OR 'heart attacks' OR 'myocardial infarct' OR 'infarct, myocardial' OR 'infarcts, myocardial' OR 'myocardial infarcts' OR 'heart infarction' OR 'myocardial infarction, inferior wall' OR 'diaphragmatic myocardial infarction' OR 'diaphragmatic myocardial infarctions' OR 'infarction, diaphragmatic myocardial' OR 'infarctions, diaphragmatic myocardial' OR 'myocardial infarction, diaphragmatic' OR 'myocardial infarctions, diaphragmatic' OR 'inferior myocardial infarction' OR 'infarction, inferior myocardial' OR 'infarctions, inferior myocardial' OR 'inferior myocardial infarctions' OR 'myocardial infarction, inferior' OR 'myocardial infarctions, inferior' OR 'acute inferior myocardial infarction' OR 'painless myocardial infarction' OR 'silent myocardial infarction' OR 'myocardial infarction, anterior wall' OR 'anterolateral myocardial infarction' OR 'anterolateral myocardial infarctions' OR 'infarction, anterolateral myocardial' OR 'infarctions, anterolateral myocardial' OR 'myocardial infarction, anterolateral' OR 'myocardial infarctions, anterolateral' OR 'anteroseptal myocardial infarction' OR 'anteroseptal myocardial infarctions' OR 'infarction, anteroseptal myocardial' OR 'infarctions, anteroseptal myocardial' OR 'myocardial infarction, anteroseptal' OR 'myocardial infarctions, anteroseptal' OR 'acute anterior wall myocardial infarction' OR 'posterior myocardial infarction' OR 'posterior wall myocardial infarction' OR 'posterior myocardial infarction' OR 'non st elevated myocardial infarction' OR 'nstemi' OR 'non-st-elevation myocardial infarction' OR 'infarction, non-st-elevation myocardial' OR 'infarctions, non-st-elevation myocardial' OR 'myocardial infarction, non-st-elevation' OR 'myocardial infarctions, non-st-elevation' OR 'non st elevation myocardial infarction' OR 'non-st-elevation myocardial infarctions' OR 'non st segment elevation myocardial infarction' OR 'non st segment elevated myocardial infarction' OR 'st segment elevation myocardial infarction' OR 'st elevated myocardial infarction' OR 'stemi' OR 'st segment elevated myocardial infarction' OR non AND 'stemi') OR 'hyperlipidemia'/exp OR 'dyslipidemia'/exp OR 'hyperlipidemias' OR 'hyperlipemia' OR 'hyperlipemias' OR 'hyperlipidemia' OR 'lipidemia' OR 'lipidemias' OR 'lipemia' OR 'lipemias' OR 'dyslipidemia' OR 'lipidemia, dys' OR 'oxidized low density lipoprotein'/exp OR 'apolipoprotein b'/exp OR 'low density lipoprotein cholesterol'/exp OR 'low density lipoprotein'/exp OR 'very low density lipoprotein'/exp OR 'very low density lipoprotein cholesterol'/exp OR 'oxidized low density lipoprotein' OR 'oxidized ldl' OR 'oxldl' OR 'ox-ldl' OR 'electronegative low-density lipoprotein' OR 'ldl' OR 'minimally modified oxidized-ldl' OR 'mm-ldl' OR 'apo low density lipoprotein' OR 'low density lipoprotein triglyceride' OR 'triacylglycerol ldl' OR 'ldl triacylglycerol' OR 'lipoprotein, ldl triglyceride' OR 'ldl triglyceride' OR 'very low density lipoprotein triglyceride' OR 'vldl triglyceride' OR 'triglyceride vldl' OR 'vldl triacylglycerol' OR 'lipoprotein, vldl triglyceride' OR 'triacylglycerol vldl' OR 'low density lipoprotein' OR 'apolipoprotein b' OR 'apo ldl' OR 'cholesterol, ldl' OR 'low density lipoprotein cholesterol' OR 'ldl cholesterol' OR 'lipoproteins, ldl' OR 'very low density lipoprotein' OR 'lipoprotein, low density' OR 'lipoproteins, ldl cholesterol' OR 'triacylglycerol'/exp OR 'triacylglycerol blood level'/exp OR 'fatty acid'/exp OR 'hypertriglyceridemia'/exp OR 'long chain triacylglycerol'/exp OR 'medium chain triacylglycerol'/exp OR 'triglycerides' OR 'triacylglycerol' OR 'triacylglycerols' OR 'triglyceride' OR 'blood triglyceride' OR 'triacylglycerol blood level' OR 'fatty acid triglyceride' OR 'long chain triglyceride' OR 'long chain triacylglycerol' OR 'medium chain triglyceride' OR 'plasma triglyceride' OR 'serum triglyceride' OR 'triglyceride blood level' OR 'triglyceride fatty acid' OR 'triglyceride plasma level' OR 'hypertriglyceridemia' OR 'triglyceride storage disease' OR 'triglyceride, long chain' OR 'triglyceride, medium chain' OR 'medium chain triacylglycerol' OR 'cholesterol'/exp OR 'cholesterol derivative'/exp OR 'cholesterol blood level'/exp OR 'disorders of cholesterol metabolism'/exp OR 'high density lipoprotein cholesterol'/exp OR 'cholesterol metabolism'/exp OR 'cholesterol, vldl' OR 'vldl cholesterol' OR 'cholesterol' OR 'pre-beta-lipoprotein cholesterol' OR 'cholesterol, pre-beta-lipoprotein' OR 'pre beta lipoprotein cholesterol' OR 'very low density lipoprotein cholesterol' OR 'prebetalipoprotein cholesterol' OR 'cholesterol, prebetalipoprotein' OR 'epicholesterol' OR 'cholesterin' OR 'cholesterol derivative' OR 'beta cholesterol' OR 'blood cholesterol' OR 'cholesterol blood level' OR 'blood cholesterol level' OR 'disorders of cholesterol metabolism' OR 'hdl cholesterol' OR 'high density lipoprotein cholesterol' OR 'cholesterol, hdl' OR 'metabolism, cholesterol' OR 'cholesterol metabolism' OR 'total blood cholesterol' OR 'total cholesterol' OR 'atherosclerosis'/exp OR 'aorta atherosclerosis'/exp OR 'peripheral occlusive artery disease'/exp OR 'coronary artery atherosclerosis'/exp OR 'carotid atherosclerosis'/exp OR 'coronary artery disease'/exp OR 'carotid artery diseases' OR 'atherosis' OR 'atherosclerosis' OR 'coronary artery disease' OR 'atheroscleroses' OR 'atherogenesis' OR 'artery disease, coronary' OR 'artery diseases, coronary' OR 'coronary artery diseases' OR 'disease, coronary artery' OR 'diseases, coronary artery' OR 'coronary arteriosclerosis' OR 'arterioscleroses, coronary' OR 'coronary arterioscleroses' OR 'atheroscleroses, coronary' OR 'coronary atheroscleroses' OR 'coronary atherosclerosis' OR 'arteriosclerosis, coronary' OR 'artery disease, carotid' OR 'artery diseases, carotid' OR 'carotid artery disease' OR 'carotid artery disorders' OR 'artery disorder, carotid' OR 'artery disorders, carotid' OR 'carotid artery disorder' OR 'aorta atherosclerosis' OR 'aortic atherosclerosis' OR 'peripheral occlusive artery disease' OR 'atherosclerosis obliterans' OR 'atherosclerosis, aorta' OR 'atherosclerosis, coronary' OR 'coronary artery atherosclerosis' OR 'atherosclerosis, coronary artery' OR 'carotid artery atherosclerosis' OR 'carotid atherosclerosis' OR 'heart atherosclerosis' OR 'obliterating atherosclerosis' OR 'coronary artery occlusive disease'

**AND**

'trimethylamine oxide' OR 'trimethylamine n oxide' OR 'trimethylaminooxide'/exp OR 'trimethylaminooxide' OR 'trimethylaminoxide'/exp OR 'trimethylaminoxide' OR 'tmao' OR 'trimethyloxamine'/exp OR 'trimethyloxamine' OR 'trimethylammonium oxide' OR 'trimethylamine n-oxide'/exp OR 'trimethylamine n-oxide'

1. **Cochrance Library search**

#1 TMAO (Word variations have been searched)

#2 trimethylamine n-oxide (Word variations have been searched)

#3 trimethylamine oxide (Word variations have been searched)

#4 trimethylamine n oxide (Word variations have been searched)

#5 trimethylaminooxide (Word variations have been searched)

#6 trimethylaminoxide (Word variations have been searched)

#7 trimethyloxamine (Word variations have been searched)

#8 trimethylammonium oxide (Word variations have been searched)

#9 trimethylamine N-oxide (Word variations have been searched)

#10 #1 or #2 or #3 or #4 or #5 or #6 or #7 or #8 or #9

1. **Web of science search**

TOPIC: (trimethylamine n-oxide) OR TOPIC: (trimethylamine oxide) OR TOPIC: (trimethylamine n oxide) OR TOPIC: (trimethylaminooxide) OR TOPIC: (trimethylaminoxide) OR TOPIC: (TMAO)

**AND**

TOPIC: (CHD) OR TOPIC: (Coronary Disease*) OR TOPIC: (Coronary Heart Disease) OR TOPIC: (ischemic heart disease) OR TOPIC: (Myocardial Infarction*) OR TOPIC: (Infarction, Myocardial) OR TOPIC: (Cardiovascular Disease*) OR TOPIC: (cardiovascular disorder) OR TOPIC: (Heart*) OR TOPIC: (cardiac*) OR TOPIC: (cardio*) OR TOPIC: (acute coronary syndrome*) OR TOPIC: (Hyperlipidemias) OR TOPIC: (oxidized low density lipoprotein) OR TOPIC: (LDL) OR TOPIC: (Triglyceride*) OR TOPIC: (Cholesterol) OR TOPIC: (Atherosclerosis) OR TOPIC: (Coronary Artery Disease) OR TOPIC: (Coronary Arteriosclerosis)

[excluding] DOCUMENT TYPES: ( REVIEW )

1. **ClinicalTrials.gov search**

"trimethyloxamine" OR "trimethylammonium oxide" OR "trimethylamine N-oxide" OR "TMAO OR trimethylamine oxide"

1. **VIP search**

'trimethylamine oxide'

1. **CNKI search**

‘angina’ OR ‘Coronary Disease’ OR ‘coronary artery disease’ OR ‘Acute Coronary Syndrome’ OR ‘atherosclerosis’

**AND**

'trimethylamine oxide'

**secondary screening:** ‘Cardiovascular Disease’

1. **SinoMed search**

'trimethylamine oxide'

1. **Wanfang Databases search**

**Title or Key word/** **Abstracts**

‘angina’ OR ‘Coronary Disease’ OR ‘coronary artery disease’ OR ‘Acute Coronary Syndrome’ OR ‘atherosclerosis’

**AND**

'trimethylamine oxide’
