# Supplementary material for: Trimethylamine-N-oxide has prognostic value in coronary heart disease: a meta-analysis and dose-response analysis
Source: BMC Cardiovasc Disord. 2020 Jan 9;20:7. doi: 10.1186/s12872-019-01310-5 (PMC6953212; doi:10.1186/s12872-019-01310-5)
Supplement: Supplementary file 2 — Additional file 2. MOOSE Checklist. [file 12872_2019_1310_MOESM2_ESM.docx]

**Additional file 2. MOOSE Checklist.**

| **Item No** | **Recommendation** | **Reported on Page No.** |
| --- | --- | --- |
| Reporting of background should include | | |
| 1 | Problem definition | 5-6 |
| 2 | Hypothesis statement | 5-6 |
| 3 | Description of study outcome(s) | 5-6 |
| 4 | Type of exposure or intervention used | 5-6 |
| 5 | Type of study designs used | 5-6 |
| 6 | Study population | 5-6 |
| Reporting of search strategy should include | | |
| 7 | Qualifications of searchers (eg, librarians and investigators) | 6 |
| 8 | Search strategy, including time period included in the synthesis and key words | 6 |
| 9 | Effort to include all available studies, including contact with authors | 7-8 |
| 10 | Databases and registries searched | 6 |
| 11 | Search software used, name and version, including special features used (eg, explosion) | NoteExpress |
| 12 | Use of hand searching (eg, reference lists of obtained articles) | 10 |
| 13 | List of citations located and those excluded, including justification | Supplemental Digital Content 5 |
| 14 | Method of addressing articles published in languages other than English | included all languages |
| 15 | Method of handling abstracts and unpublished studies | 10 |
| 16 | Description of any contact with authors | 7-8 |
| Reporting of methods should include | | |
| 17 | Description of relevance or appropriateness of studies assembled for assessing the hypothesis to be tested | 6-7 |
| 18 | Rationale for the selection and coding of data (eg, sound clinical principles or convenience) | 7-8 |
| 19 | Documentation of how data were classified and coded (eg, multiple raters, blinding and interrater reliability) | 8 |
| 20 | Assessment of confounding (eg, comparability of cases and controls in studies where appropriate) | 7-8 |
| 21 | Assessment of study quality, including blinding of quality assessors, stratification or regression on possible predictors of study results | 8 |
| 22 | Assessment of heterogeneity | 9 |
| 23 | Description of statistical methods (eg, complete description of fixed or random effects models, justification of whether the chosen models account for predictors of study results, dose-response models, or cumulative meta-analysis) in sufficient detail to be replicated | 8-9, Supplemental Digital Content 4 |
| 24 | Provision of appropriate tables and graphics | Figures 1-3, Table 1, Supplemental Digital Content |
| Reporting of results should include | | |
| 25 | Graphic summarizing individual study estimates and overall estimate | Figures 1-3 |
| 26 | Table giving descriptive information for each study included | Table 1 |
| 27 | Results of sensitivity testing (eg, subgroup analysis) | 12-13, Supplemental Digital Content 6 |
| 28 | Indication of statistical uncertainty of findings | 17-18 |

*From*:

Stroup DF, Berlin JA, Morton SC, Olkin I, Williamson GD, Rennie D, et al. Meta-analysis of observational studies in epidemiology: a proposal for reporting. Meta-analysis of observational studies in epidemiology (MOOSE) group. *JAMA* 2000; **283**:2008-2012.
