# Supplementary material for: Trimethylamine-N-oxide has prognostic value in coronary heart disease: a meta-analysis and dose-response analysis
Source: BMC Cardiovasc Disord. 2020 Jan 9;20:7. doi: 10.1186/s12872-019-01310-5 (PMC6953212; doi:10.1186/s12872-019-01310-5)
Supplement: Supplementary file 3 — Additional file 3. Data used in the ‘TMAO concentration-risk of MACE’ analysis. [file 12872_2019_1310_MOESM3_ESM.docx]

**Additional file 3. Data used in the ‘TMAO concentration-risk of MACE’ analysis.**

|  | **Study** | **TMAO ^a^** | **Interval** | **N** | **Case** | **Control** | **HR** | **Lb** | **Ub** |
| --- | --- | --- | --- | --- | --- | --- | --- | --- | --- |
| **1** | CC Li XS 2017 **[9]** | 1.335 | 0.12-2.55 | 119 | 18 | 101 | 1 | 1 | 1 |
|  |  | 3.415 | 2.56-4.27 | 118 | 22 | 96 | 0.88 | 0.554 **^b^** | 1.399 **^b^** |
|  |  | 6.085 | 4.28-7.89 | 118 | 39 | 79 | 1.43 | 0.974 **^b^** | 2.099 **^b^** |
|  |  | 9.492 | 7.91-186.10 | 119 | 57 | 62 | 1.81 | 1.04 | 3.15 |
| **2** | SC Li XS 2017 **[9]** | 1.005 | 0.08-1.93 | 421 | 211 | 210 | 1 | 1 | 1 |
|  |  | 2.4 | 1.94-2.86 | 418 | 201 | 217 | 0.81 | 0.669 **^b^** | 0.981 **^b^** |
|  |  | 3.855 | 2.87-4.84 | 420 | 203 | 217 | 1.02 | 0.842 **^b^** | 1.235 **^b^** |
|  |  | 5.82 | 4.85-246.6 | 424 | 217 | 207 | 1.57 | 1.03 | 2.41 |
| **3** | Tang WH 2013 **[5]** | 1.215 | <2.43 | 1001 | 174 | 827 | 1 | 1 | 1 |
|  |  | 3.045 | 2.43-3.66 | 998 | 155 | 843 | 1.06 | 0.77 | 1.45 |
|  |  | 4.925 | 3.67-6.18 | 1003 | 168 | 835 | 1.11 | 0.82 | 1.51 |
|  |  | 7.416 | >6.18 | 1005 | 214 | 791 | 1.43 | 1.05 | 1.94 |

**Case**, the numbers of patients with major adverse cardiovascular events; **CC**, Cleveland acute coronary syndrome cohort; **Control**, the numbers of patients without MACE; **HR**, hazard ratios for MACEs in every level of TMAO; **Interval**, the range of TMAO in every level; **Lb**, lower limit of HRs; **N**, the total numbers of patients; **MACE,** major adverse cardiovascular events; **SC**, Swiss ACS cohort; **TMAO**, trimethylamine-N-oxide; **Ub**, upper limit of HRs.

**^a^** The midpoint of TMAO in each category of interval. Particularly, when the interval was an open-ending, TMAO was calculated by the lowest limit multiplying by 1.2.

**^b^** Data were calculated by a HR meta-analysis spreadsheet developed by Hans Messersmith.
