# Supplementary material for: Trimethylamine-N-oxide has prognostic value in coronary heart disease: a meta-analysis and dose-response analysis
Source: BMC Cardiovasc Disord. 2020 Jan 9;20:7. doi: 10.1186/s12872-019-01310-5 (PMC6953212; doi:10.1186/s12872-019-01310-5)
Supplement: Supplementary file 4 — Additional file 4. Dose-response analysis by Stata/SE 14.0 (commands). [file 12872_2019_1310_MOESM4_ESM.docx]

**Additional file 4. Dose-response analysis by Stata/SE 14.0 (commands).**

. gen loghr=ln( hr )

. gen seloghr=(ln( ub )-ln( lb ))/(2* invnormal(.975))

. bysort id :gen tmaoc = tmao- tmao[1]

. mkspline tmaocs =tmaoc, nk(3) cubic

. mkspline tmaos = tmao, nk(3) cubic

. mvmeta_make glst loghr tmaocs1 tmaocs2, cov( n case ) se( seloghr ) pfirst( id studyt) saving( ssest_spline ) replace by( id ) names( b V )

preserve

. use ssest_spline.dta, clear

. mvmeta b V, mm i2

testparm btmaocs2

capture estimates save mvmeta, replace

. restore

. estimates use mvmeta

summarize tmaos1 tmaos2 if tmao

predictnl loghr_sp = _b[btmaocs1]*(tmaos1-4.17)+_b[btmaocs2]*(tmaos2-1.14),ci(low up)

gen hrs = exp(loghr_sp)

gen lbs = exp(low)

gen ubs = exp(up)

glst loghr tmaoc, cov(n case) se(seloghr) pfirst(id studyt) ts(r)

predictnl loghr_line = _b[tmaoc]*(tmao-4.17)

gen hrl = exp(loghr_line)

twoway (line hrl tmao, sort lc(black) lp(-)) (line hrs lbs ubs tmao, sort lw(thick thick thick) lc(black black black) lp(l longdash longdash)), yscale(log) ytitle(“Hazard Ratio”) xtitle (“Trimetlylamine Oxide”) scheme(s1mono) xlabel(0(1)10) xmtick(0(.1)10) ymtick(0(.1)2) ylabel(1 2 3,format(%3.2fc) angle(horiz)) legend(label(1 “Linear Model”) label(2 “Spline Model”) order(1 2) ring(0) pos(11) col(1) ) plotregion(style(none))
