# Supplementary material for: Trimethylamine-N-oxide has prognostic value in coronary heart disease: a meta-analysis and dose-response analysis
Source: BMC Cardiovasc Disord. 2020 Jan 9;20:7. doi: 10.1186/s12872-019-01310-5 (PMC6953212; doi:10.1186/s12872-019-01310-5)
Supplement: Supplementary file 5 — Additional file 5. Summary of 57 disqualified papers by evaluating full texts. [file 12872_2019_1310_MOESM5_ESM.docx]

**Additional file 5. Summary of 57 disqualified papers by evaluating full texts.**

| **NO.** | **Reference** |
| --- | --- |
| **Patients without CHD at baseline (23 papers)** | |
| 1 | 42. Kim RB, Morse BL, Djurdjev O, Tang M, Muirhead N, Barrett B, et al. Advanced chronic kidney disease populations have elevated trimethylamine N-oxide levels associated with increased cardiovascular events. Kidney Int. 2016;89:1144-52. |
| 2 | 43. Trøseid M, Hov JR, Nestvold TK, Thoresen H, Berge RK, Svardal A, et al. Major increase in microbiota-dependent proatherogenic metabolite TMAO one year after bariatric surgery. Metab Syndr Relat Disord. 2016;14:197-201. |
| 3 | 44. Miller PE, Haberlen SA, Brown TT, Margolick JB, DiDonato JA, Hazen SL, et al. Brief report: intestinal microbiota-produced trimethylamine-N-oxide and its association with coronary stenosis and HIV serostatus. J Acquir Immune Defic Syndr. 2016;72:114-8. |
| 4 | 45. Haissman JM, Knudsen A, Hoel H, Kjær A, Kristoffersen US, Berge RK, et al. Microbiota-dependent marker TMAO is elevated in silent ischemia but is not associated with first-time myocardial infarction in HIV infection. J Acquir Immune Defic Syndr. 2016;71:130-6. |
| 5 | 46. Stubbs JR, House JA, Ocque AJ, Zhang S, Johnson C, Kimber C, et al. Serum trimethylamine-N-oxide is elevated in CKD and correlates with coronary atherosclerosis burden. J Am Soc Nephrol. 2016;27:305-13. |
| 6 | 47. Kaysen GA, Johansen KL, Chertow GM, Dalrymple LS, Kornak J, Grimes B, et al. Associations of trimethylamine N-oxide with nutritional and inflammatory biomarkers and cardiovascular outcomes in patients new to dialysis. J Ren Nutr. 2015;25:351-6. |
| 7 | 48. Fukami K, Yamagishi S, Sakai K, Kaida Y, Yokoro M, Ueda S, et al. Oral L-carnitine supplementation increases trimethylamine-N-oxide but reduces markers of vascular injury in hemodialysis patients. J Cardiovasc Pharmacol. 2015;65:289-95. |
| 8 | 49. Tang WH, Wang Z, Shrestha K, Borowski AG, Wu Y, Troughton RW, et al. Intestinal microbiota-dependent phosphatidylcholine metabolites, diastolic dysfunction, and adverse clinical outcomes in chronic systolic heart failure. J Card Fail. 2015;21:91-6. |
| 9 | 50. Tang WH, Wang Z, Fan Y, Levison B, Hazen JE, Donahue LM, et al. Prognostic value of elevated levels of intestinal microbe-generated metabolite trimethylamine-N-oxide in patients with heart failure: refining the gut hypothesis. J Am Coll Cardiol. 2014;64:1908-14. |
| 10 | 51. Lewis GD, Wei R, Liu E, Yang E, Shi X, Martinovic M, et al. Metabolite profiling of blood from individuals undergoing planned myocardial infarction reveals early markers of myocardial injury. J Clin Invest. 2008;118:3503-12. |
| 11 | 52. Knudsen A, Christensen TE, Thorsteinsson K, Ghotbi AA, Hasbak P, Lebech AM, et al. Microbiota-dependent marker TMAO is not associated with decreased myocardial perfusion in well-treated HIV-infected patients as assessed by 82rubidium PET/CT. J Acquir Immune Defic Syndr. 2016;72:e83-5. |
| 12 | 53. Suzuki T, Heaney LM, Bhandari SS, Jones DJ, Ng LL. Trimethylamine N-oxide and prognosis in acute heart failure. Heart. 2016;102:841-8. |
| 13 | 54. Dambrova M, Latkovskis G, Kuka J, Strele I, Konrade I, Grinberga S, et al. Diabetes is associated with higher trimethylamine N-oxide plasma levels. Exp Clin Endocrinol Diabetes. 2016;124:251-6. |
| 14 | 55. Meyer KA, Benton TZ, Bennett BJ, Jacobs DR Jr, Lloyd-Jones DM, Gross MD, et al. Microbiota-dependent metabolite trimethylamine N-oxide and coronary artery calcium in the coronary artery risk development in young adults study (CARDIA). J Am Heart Assoc. 2016;5:e003970. |
| 15 | 56. Senthong V, Wang Z, Fan Y, Wu Y, Hazen SL, Tang WH. Trimethylamine N-oxide and mortality risk in patients with peripheral artery disease. J Am Heart Assoc. 2016;5:e004237. |
| 16 | 57. Tang WH, Wang Z, Fan Y, Levison B, Hazen JE, Donahue LM, et al. Prognostic value of elevated levels of intestinal microbe-generated metabolite trimethylamine-N-oxide in patients with heart failure: refining the gut hypothesis. J Am Coll Cardiol. 2014;64:1908-14 |
| 17 | 58. Shafi T, Powe NR, Meyer TW, Hwang S, Hai X, Melamed ML, et al. Trimethylamine N-Oxide and cardiovascular events in hemodialysis patients. J Am Soc Nephrol. 2017;28:321-31. |
| 18 | 59. Hove-Skovsgaard M, Gaardbo JC, Kolte L, Winding K, Seljeflot I, Svardal A, et al. HIV-infected persons with type 2 diabetes show evidence of endothelial dysfunction and increased inflammation. BMC Infect Dis. 2017;17:234. |
| 19 | 60. Svingen GFT, Zuo H, Ueland PM, Seifert R, Løland KH, Pedersen ER, et al. Increased plasma trimethylamine-N-oxide is associated with incident atrial fibrillation. Int J Cardiol. 2018;267:100-106. |
| 20 | 61. Winther SA, Øllgaard JC, Tofte N, Tarnow L, Wang Z, Ahluwalia TS, et al. Utility of Plasma Concentration of Trimethylamine N-Oxide in Predicting Cardiovascular and Renal Complications in Individuals With Type 1 Diabetes. Diabetes Care. 2019;42:1512-1520. |
| 21 | 62. Stubbs JR, Stedman MR, Liu S, Long J, Franchetti Y, West RE 3rd, et al. Trimethylamine N-Oxide and Cardiovascular Outcomes in Patients with End-stage Kidney Disease Receiving Maintenance Hemodialysis. Clin J Am Soc Nephrol. 2019;pii:CJN.06190518. |
| 22 | 63. Zhai Q, Wang X, Chen C, Tang Y, Wang Y, Tian J, et al. Prognostic Value of Plasma Trimethylamine N-Oxide Levels in Patients with Acute Ischemic Stroke. Cell Mol Neurobiol. 2019;39:1201-1206. |
| 23 | 64. Suzuki T, Yazaki Y, Voors AA, Jones DJL, Chan DCS, Anker SD, et al. Association with outcomes and response to treatment of trimethylamine N-oxide in heart failure: results from BIOSTAT-CHF. Eur J Heart Fail. 2019;21:877-886. |
| **No relevant outcomes data (18 papers)** | |
| 1 | 65. Mente A, Chalcraft K, Ak H, Davis AD, Lonn E, Miller R, et al. The relationship between trimethylamine-N-oxide and prevalent cardiovascular disease in a multiethnic population living in Canada. Can J Cardiol. 2015;31:1189-94. |
| 2 | 66. Trøseid M, Ueland T, Hov JR, Svardal A, Gregersen I, Dahl CP, et al. Microbiota-dependent metabolite trimethylamine-N-oxide is associated with disease severity and survival of patients with chronic heart failure. J Intern Med. 2015;277:717-26. |
| 3 | 67. Kühn T, Rohrmann S, Sookthai D, Johnson T, Katzke V, Kaaks R, et al. Intra-individual variation of plasma trimethylamine-N-oxide (TMAO), betaine and choline over 1 year. Clin Chem Lab Med. 2017;55:261-8. |
| 4 | 68. Fu Q, Zhao M, Wang D, Hu H, Guo C, Chen W, et al. Coronary plaque characterization assessed by optical coherence tomography and plasma trimethylamine-N-oxide levels in patients with coronary artery disease. Am J Cardiol. 2016;118:1311-5. |
| 5 | 69. Mafune A, Iwamoto T, Tsutsumi Y, Nakashima A, Yamamoto I, Yokoyama K, et al. Associations among serum trimethylamine-N-oxide (TMAO) levels, kidney function and infarcted coronary artery number in patients undergoing cardiovascular surgery: a cross-sectional study. Clin Exp Nephrol. 2016;20:731-9. |
| 6 | 70. Senthong V, Li XS, Hudec T, Coughlin J, Wu Y, Levison B, et al. Plasma trimethylamine N-Oxide, a gut microbe-generated phosphatidylcholine metabolite, is associated with atherosclerotic burden. J Am Coll Cardiol. 2016;67:2620-8. |
| 7 | 71. Miller PE, Haberlen SA, Brown TT, Margolick JB, DiDonato JA, Hazen SL, et al. Brief report: intestinal microbiota-produced trimethylamine-N-oxide and its association with coronary stenosis and HIV serostatus. J Acquir Immune Defic Syndr. 2016;72:114-8. |
| 8 | 72. Zheng Y, Li Y, Rimm EB, Hu FB, Albert CM, Rexrode KM, et al. Dietary phosphatidylcholine and risk of all-cause and cardiovascular-specific mortality among US women and men. Am J Clin Nutr. 2016;104:173-80. |
| 9 | 73. Liu X, Xie Z, Sun M, Wang X, Li J, Cui J, et al. Plasma trimethylamine N-oxide is associated with vulnerable plaque characteristics in CAD patients as assessed by optical coherence tomography. Int J Cardiol. 2018;265:18-23. |
| 10 | 74. Dong Z, Liang Z, Guo M, Hu S, Shen Z, Hai X. The Association between Plasma Levels of Trimethylamine N-Oxide and the Risk of Coronary Heart Disease in Chinese Patients with or without Type 2 Diabetes Mellitus. Dis Markers. 2018;2018:1578320. |
| 11 | 75. Żurawska-Płaksej E, Płaczkowska S, Pawlik-Sobecka L, Czapor-Irzabek H, Stachurska A, Mysiak A, et al. Parameters of Oxidative and Inflammatory Status in a Three-Month Observation of Patients with Acute Myocardial Infarction Undergoing Coronary Angioplasty-A Preliminary Study. Medicina (Kaunas). 2019;55:pii: E585. |
| 12 | 76. Li XS, Obeid S, Wang Z, Hazen BJ, Li L, Wu Y, et al. Trimethyllysine, a trimethylamine N-oxide precursor, provides near- and long-term prognostic value in patients presenting with acute coronary syndromes. Eur Heart J. 2019;40:2700-2709. |
| 13 | 77. Sheng Z, Tan Y, Liu C, Zhou P, Li J, Zhou J, et al. Relation of Circulating Trimethylamine N-Oxide With Coronary Atherosclerotic Burden in Patients With ST-segment Elevation Myocardial Infarction. Am J Cardiol. 2019;123:894-898. |
| 14 | 78. Chou RH, Chen CY, Chen IC, Huang HL, Lu YW, Kuo CS, et al. Trimethylamine N-Oxide, Circulating Endothelial Progenitor Cells, and Endothelial Function in Patients with Stable Angina. Sci Rep. 2019;9:4249. |
| 15 | 79. Zhong Z, Liu J, Zhang Q, Zhong W, Li B, Li C, et al. Targeted metabolomic analysis of plasma metabolites in patients with coronary heart disease in southern China. Medicine (Baltimore). 2019;98:e14309. |
| 16 | 80. Tan Y, Sheng Z, Zhou P, Liu C, Zhao H, Song L, et al. Plasma Trimethylamine N-Oxide as a Novel Biomarker for Plaque Rupture in Patients With ST-Segment-Elevation Myocardial Infarction. Circ Cardiovasc Interv. 2019;12:e007281. |
| 17 | 81. Tarik Alhmouda, Anand Kumarb, Chien-Chi Lob, Rana Al-Sadia, Stacey Clegga, Ihab Alomaric, et al. Investigating intestinal permeability and gut microbiota roles in acute coronary syndrome patients. Human Microbiome Journal. 2019;doi:10.1016/j.humic.2019.100059. |
| 18 | 82. Yuan X-Y, Pan M, Yang P, Chen C, Shi W-H, Yuan J-P, et al. Evaluation value of plasma trimethylamine oxide determination for ventricular remodeling and prognosis in patients with acute myocardial infarction. Shandong Medical Journal. 2019;59:10-13. |
| **Unrelated topic (3 papers)** | |
| 1 | 83. Koeth RA, Wang Z, Levison BS, Buffa JA, Org E, Sheehy BT, et al. Intestinal microbiota metabolism of L-carnitine, a nutrient in red meat, promotes atherosclerosis. Nat Med. 2013;19:576-85. |
| 2 | 84. Dambrova M, Skapare-Makarova E, Konrade I, Pugovics O, Grinberga S, Tirzite D, et al. Meldonium decreases the diet-increased plasma levels of trimethylamine N-oxide, a metabolite associated with atherosclerosis. J Clin Pharmacol. 2013;53:1095-8. |
| 3 | 85. Randrianarisoa E, Lehn-Stefan A, Wang X, Hoene M, Peter A, Heinzmann SS, et al. Relationship of serum trimethylamine N-oxide (TMAO) levels with early atherosclerosis in humans. Sci Rep. 2016;6:26745. |
| **Meeting abstracts (5 papers)** | |
| 1 | 86. Velebova K, Hoang T, Veleba J, Belinova L, Kopecky J, Kuda O, et al. The effect of metformin on serum levels of trimethylamine-N-oxide in patients with type 2 diabetes/prediabetes and chronic heart failure. Diabetologia. 2016;59:S533. |
| 2 | 87. Senthong V, Wang Z, Wu Y, Tang WH, Hazen S. Elevated plasma trimethylamine-N-oxide is associated with poor prognosis in patients with peripheral artery disease. J Am Coll Cardiol. 2015;65:A2076. |
| 3 | 88. Zhu W, Wang Z, Org E, Gupta N, Silverstein RL, McIntyre TM, et al. Gut flora-depend metabolite, TMAO, promotes thrombosis. Cardiovasc Pathol. 2014;23:e26-7. |
| 4 | 89. Shrestha K, Wang Z, Borowski AG, Troughton RW, Klein AL, Hazen S, et al. Gut-flora-dependent phosphatidylcholine metabolites predict adverse clinical outcomes in chronic systolic heart failure. J Card Fail. 2011;17:S28. |
| 5 | 90. Tang WHW, Wang ZN, Wu YP, Fan YY, Koeth RA, Hazen S. Gut flora metabolite trimethylamine N-oxide predicts incident cardiovascular risks in both stable non-diabetics and diabetic subjects. J Am Coll Cardiol. 2013;61:E1398. |
| **Reviews (6 papers)** | |
| 1 | 30. Zhu W, Gregory JC, Org E, Buffa JA, Gupta N, Wang Z, et al. Gut microbial metabolite TMAO enhances platelet hyperreactivity and thrombosis risk. Cell. 2016;165:111-24. |
| 2 | 91. Martínez-Del Campo A, Romano KA, Rey FE, Balskus EP. The plot thickens: diet microbe interactions may modulate thrombosis risk. Cell Metab. 2016;23:573-5. |
| 3 | 92. Yamashina A. Gut microbiota and internal diseases: update information. Topics: V. Gut Microbiota: Topics in Various Medical Fields; 1. Does intestinal flora promote atherosclerosis?. Nihon Naika Gakkai Zasshi. 2015;104:66-70. |
| 4 | 93. Obin M, Parnell LD, Ordovas JM. The emerging relevance of the gut microbiome in cardiometabolic health. Curr Cardiovasc Risk Rep. 2013;7. |
| 5 | 94. Sitaraman R. Phospholipid catabolism by gut microbiota and the risk of cardiovascular disease. J Med Microbiol. 2013;62:948-50. |
| 6 | 95. Gut microbes may affect heart disease risk. But studies in rodents suggesting a link may not play out in people. Harv Heart Lett. 2012;22:6. |
| **Insufficient information on the inclusion criterion and follow-up period (1 papers)** | |
| 1 | 24. Wang Z, Klipfell E, Bennett BJ, Koeth R, Levison BS, Dugar B, et al. Gut flora metabolism of phosphatidylcholine promotes cardiovascular disease. Nature. 2011;472:57-63. |
| **A repetitive report from a partial dataset (1 papers)** | |
| 1 | 25. Wang Z, Tang WH, Buffa JA, Fu X, Britt EB, Koeth RA, et al. Prognostic value of choline and betaine depends on intestinal microbiota-generated metabolite trimethylamine-N-oxide. Eur Heart J. 2014;35:904-10. |
