# Supplementary material for: Trimethylamine-N-oxide has prognostic value in coronary heart disease: a meta-analysis and dose-response analysis
Source: BMC Cardiovasc Disord. 2020 Jan 9;20:7. doi: 10.1186/s12872-019-01310-5 (PMC6953212; doi:10.1186/s12872-019-01310-5)
Supplement: Supplementary file 6 — Additional file 6. Summarized results on the relative risk of MACE of elevated TMAO in patients with CHD. [file 12872_2019_1310_MOESM6_ESM.docx]

**Additional file 6. Summarized results on the relative risk of MACE of elevated TMAO in patients with CHD.**

| **Study types** | **Number**  **of studies** | **Pooled HR**  **(random-effects)** | | **Heterogeneity(fixed-effects)** | | **Sensitivity analysis ^a^** | |
| --- | --- | --- | --- | --- | --- | --- | --- |
|  |  | **95% CI** | **P value** | **I^2^(%)** | **P_Q-test_** | **Fixed-effects**  **pooled HR(95%CI)** | **Reverse correlation** |
| **All** | 9 | 1.58 (1.35-1.84) | 0.000 | 57.7 | 0.015 | 1.38 (1.30-1.46) | No |
| **All (without**  **Kaizu X 2018)** | 8 | 1.47 (1.30-1.66) | 0.000 | 40.7 | 0.107 | 1.37 (1.29-1.46) |  |
| **ACS ^b^** | 7 | 1.87 (1.41-2.47) | 0.000 | 56.5 | 0.032 | 1.48 (1.35-1.63) |  |
| **ACS (** **without**  **Kaizu X 2018)** | 6 | 1.65 (1.34-2.03) | 0.000 | 31.6 | 0.199 | 1.46 (1.33-1.61) |  |
| **Chronic CHD ^b^** | 2 | 1.37 (1.11-1.70) | 0.004 | 34.4 | 0.217 | 1.31 (1.21-1.42) |  |
| **In-hospital ^c^** | 1 | 6.01 (2.03-17.76) | 0.001 | - | - | 6.01 (2.03-17.76) |  |
| **Follow-up 1-3 years ^c^** | 3 | 1.34 (1.26-1.43) | 0.000 | 0.0 | 0.414 | 1.34 (1.26-1.43) |  |
| **Follow-up ≥ 4 years ^c^** | 5 | 1.96 (1.52-2.52) | 0.000 | 0.0 | 0.764 | 1.96 (1.52-2.52) |  |

**ACS**, acute coronary syndrome; **CHD**, coronary heart diseases; **CI,** confidence interval; **HR**, hazard ratio; **I^2^**, I^2^ statistics; **MACE,** major adverse cardiovascular events; **P**, P value of Chi-squared Q-tests; **TMAO,** trimethylamine-N-oxide.

**^a^** Whether reverse outcome associations were found in the sensitivity analysis, by using different analysis models.

**^b^** The P-value of heterogeneity between these two groups was 0.058.

**^c^** P-value of heterogeneity among these three subgroups was 0.000.
